# Mutations in *NLRP5* are associated with reproductive wastage and multilocus imprinting disorders in humans

**DOI:** 10.1038/ncomms9086

**Published:** 2015-09-01

**Authors:** Louise E. Docherty, Faisal I. Rezwan, Rebecca L. Poole, Claire L. S. Turner, Emma Kivuva, Eamonn R. Maher, Sarah F. Smithson, Julian P. Hamilton-Shield, Michal Patalan, Maria Gizewska, Jaroslaw Peregud-Pogorzelski, Jasmin Beygo, Karin Buiting, Bernhard Horsthemke, Lukas Soellner, Matthias Begemann, Thomas Eggermann, Emma Baple, Sahar Mansour, I. Karen Temple, Deborah J. G. Mackay

**Affiliations:** 1 Academic Unit of Human Development and Health, Faculty of Medicine, University of Southampton, Southampton General Hospital, Southampton SO16 6YD, UK; 2 Wessex Regional Genetics Laboratory, Salisbury NHS Foundation Trust, Salisbury SP2 8BJ, UK; 3 Peninsula Clinical Genetics Service, Royal Devon and Exeter Hospital, Exeter EX1 2ED, UK; 4 Department of Medical Genetics, University of Cambridge, and Cambridge NIHR Biomedical Research Centre, Addenbrooke’s Hospital, Cambridge CP2 0QQ, UK; 5 Department of Clinical Genetics, University Hospitals Bristol, Bristol BS2 8EG, UK; 6 School of Clinical Sciences, University of Bristol, Bristol BS2 8AE, UK; 7 Department of Pediatrics, Endocrinology, Diabetology, Metabolic Diseases and Cardiology, Pomeranian Medical University, 71-252, Szczecin, Poland; 8 Department of Paediatric Oncology, Pomeranian Medical University, 71-252 Szczecin, Poland; 9 Institut für Humangenetik, Universitätsklinikum Essen, Universität Duisburg-Essen, Hufelandstr 55, 45122 Essen, Germany; 10 Institut für Humangenetik, University Hospital, RWTH Aachen, Pauwelsstr 30, 52074 Aachen, Germany; 11 Wessex Clinical Genetics Service, University Hospital Southampton NHS Foundation Trust, Southampton SO16 5YA, UK; 12 St George’s Healthcare NHS Trust, University of London, London, SW17 0QT UK

## Abstract

Human-imprinting disorders are congenital disorders of growth, development and metabolism, associated with disturbance of parent of origin-specific DNA methylation at imprinted loci across the genome. Some imprinting disorders have higher than expected prevalence of monozygotic twinning, of assisted reproductive technology among parents, and of disturbance of multiple imprinted loci, for which few causative *trans*-acting mutations have been found. Here we report mutations in *NLRP5* in five mothers of individuals affected by multilocus imprinting disturbance. Maternal-effect mutations of other human NLRP genes, *NLRP7* and *NLRP2*, cause familial biparental hydatidiform mole and multilocus imprinting disturbance, respectively. Offspring of mothers with *NLRP5* mutations have heterogenous clinical and epigenetic features, but cases include a discordant monozygotic twin pair, individuals with idiopathic developmental delay and autism, and families affected by infertility and reproductive wastage. *NLRP5* mutations suggest connections between maternal reproductive fitness, early zygotic development and genomic imprinting.

## Abstract (web summary)

Genomic imprinting disturbance can give rise to complex congenital disorders affecting growth, metabolism and behaviour. Here the authors report mutations in *NLRP5*, which suggests a connection between imprinting, maternal reproductive fitness and zygotic development.

## Graphical abstract

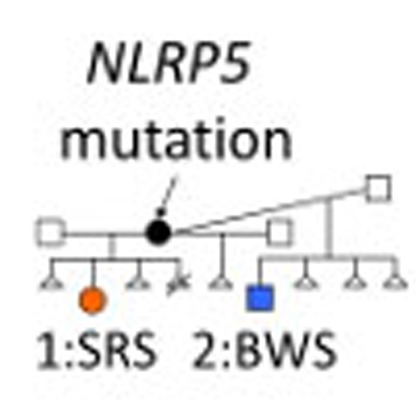

On fertilization, the genome undergoes genome-wide epigenetic reprogramming to supersede the developmental programmes of the sperm and oocyte with that of the developing zygote. A small number of gametic epigenetic marks are impervious to this reprogramming, and survive in the organism as genomic imprints, regulating genes under their control according to their parent of origin^1^. Disturbance of imprinting causes imprinting disorders affecting metabolism, growth and behaviour^2^. *Cis*-acting mutations, that is, affecting only one imprint and the gene(s) controlled by it, are associated with a range of clinically defined human-imprinting disorders, while *trans*-acting mutations may affect establishment or maintenance of multiple imprinting marks across the genome, and thus may have a wider impact on development^2^. Multilocus imprinting disturbance (MLID) is present in a minority of patients with clinically defined imprinting disorders. Mutations of *NLRP2* and *ZFP57* have been identified in rare cases of MLID^3^,^4^ but in the majority the cause is unknown.

Imprinted loci throughout the genome are paternalized in molar pregnancies; like imprinting disorders these can occur in sporadic or heritable forms, and the best-established cause of recurrent molar pregnancy is mutation of *NLRP7* or *KHDC3L*^5^,^6^,^7^. *NLRP7* and *KHDC3L* are maternal-effect genes, expressed from the maternal genome and required for zygotic development before activation of the zygotic genome^8^, such that loss of function has no effect on males or their fertility, nor on the females themselves, but females are sterile because their oocytes do not support early development. Another NLRP (nucleotide-binding domain and leucine-rich repeat-containing receptor protein) family member, *NLRP5* or its murine counterpart *Mater*, was the first-described maternal-effect gene in mice^9^.

We performed whole-exome sequencing of a large cohort of MLID patients, to seek genetic causes of the epigenetic errors in MLID. This led to the identification of NLRP5 variants in five mothers of patients with MLID. As well as offspring with MLID, though without consistent clinical presentation or consistent epimutations, some mothers with *NLRP5* variants also have periods of infertility, and reproductive outcomes including miscarriage and reported molar pregnancy. *NLRP5* is a novel candidate gene causing MLID, with deleterious maternal-effect variants in 15% of our cohort.

## Results

### *NLRP5* mutations in mothers of MLID patients

We performed whole-exome sequencing on patients with clinically defined imprinting disorders and molecular evidence of MLID, and with no known genetic cause of disorder, including eight with Beckwith–Wiedemann syndrome (BWS; MIM #130659), 10 with Silver–Russell syndrome (SRS; MIM #180860) and 7 with transient neonatal diabetes mellitus (MIM #601410). In addition, parental samples were exome sequenced for four BWS–MLID, five SRS–MLID and four transient neonatal diabetes mellitus–MLID patients, and the mother of two siblings with MLID (SRS and BWS presentations). *NLRP5* was the only gene in which rare and novel variants were found in more than two individuals. Using Sanger sequencing, *NLRP5* was sequenced in 14 further MLID patients and 19 mothers. In total, 39 MLID patients and 33 mothers were sequenced for *NLRP5* in this study (summarized in Supplementary Data 1).

**Figure 1:**
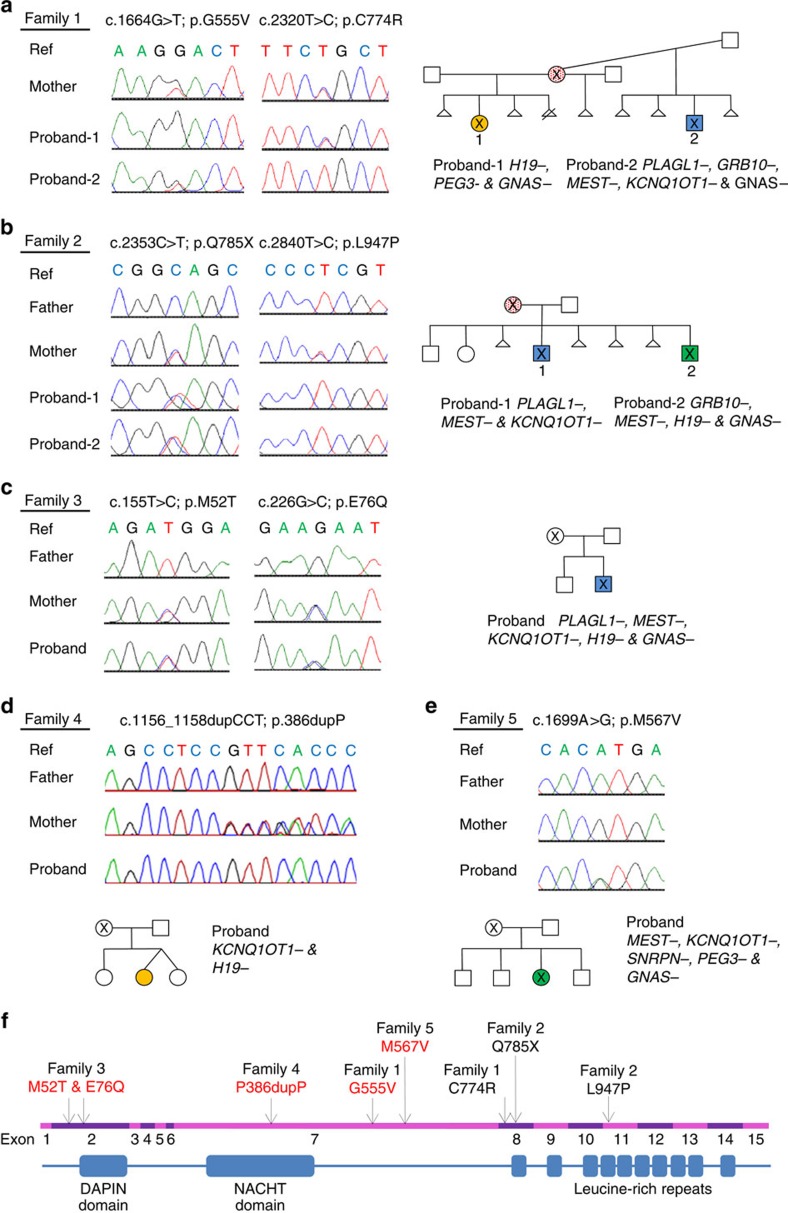
Sequence analysis of mutations in *NLRP5.* (**a**–**e**) mutations, sequencing electropherograms, pedigrees and key multilocus imprinting disturbance (MLID) imprinted loci from pedigrees 1–5. The mutation, nucleotide and amino-acid information is summarized for each pedigree, below this the wild-type sequence is provided (ref), followed by available parental genotypes and proband sequencing electropherograms. Filled symbols in pedigrees represent individuals with SRS–MLID (orange) BWS–MLID (blue) and clinically non-specific-MLID (green), red dots indicate those affected by pregnancy losses with black crosses indicating one or more *NLRP5* mutations. For each proband a list of key MLID loci are included (the — symbol indicates hypomethylation relative to controls) (**f**) Diagrammatic structure of the human NLRP5 protein showing DAPIN, NACHT and leucine-rich repeat domains is aligned to the cDNA; arrows indicate the position, variant and pedigree information of each protein alterations with novel alterations in red text.

*NLRP5* variants were detected in 5 of 33 unrelated MLID imprinting disorder pedigrees, where maternal samples were available (Fig. 1), a detection rate of 15% in our cohort. In family 1, two heterozygous single-nucleotide polymorphisms (SNPs) were identified: NM_153447.4:c.2320T>C (Cys774Arg; rs370837790; unknown minor-allele frequency) in the mother and proband 1 with SRS–MLID, and the novel variant NM_153447.4:c.1664G>T (Gly555Val) in the mother and proband 2 with BWS–MLID; neither of them was within a known functional domain. In family 2, NM_153447.4:c.2353C>T: (Gln785X; rs200446614, unknown minor-allele frequency) was found heterozygously in both affected siblings (proband 1 with BWS–MLID and proband 2 with a clinically non-specific autism and obesity–MLID) and their mother; the variant truncates NLRP5 in the first of 10 leucine-rich repeats (LRR). Sanger sequencing identified a further heterozygous variant in the mother, not inherited by either affected offspring: NM_153447.4:c.2840T>C (Leu947Pro; rs202181446; minor-allele frequency 0.15%). The mother inherited the nonsense variant from her father and the missense variant from her mother. In family 3, two novel heterozygous SNPs NM_153447.4:c.155T>C (Met52Thr) and NM_153447.4:c.226G>C (Glu76Gln) were identified, one near and one within the DAPIN (Domain in Apoptosis and INterferon response) domain. The variants were found in *cis*, being both present on individual exome sequencing reads, and present in both the proband with BWS–MLID and his mother. In family 4, a novel heterozygous duplication NM_153447.4:c.1156_1158dupCCT (386dupP) was identified within the NACHT (NAIP, CIITA, HET-E and TP1) domain. Strikingly, the duplication was found neither in the proband with SRS–MLID, nor in her twin sister, but in their mother. Sanger sequencing identified an additional novel variant in family 5, NM_153447.4:c.1699A>G (Met567Val), which was present heterozygously in the proband (clinically non-specific growth, developmental delay and unusual behaviour MLID) and homozygously in her mother. Copy number variation analysis by digital PCR confirmed there to be two copies of *NLRP5* exon 7 in the mother (Supplementary Table 1), eliminating a deletion as the cause of homozygosity for this variant. All the mothers had a normal phenotype and epigenotype, but MLID cases had overlapping imprinting disorder features often with abnormal neurodevelopment. The clinical details of each pedigree are detailed in the Methods section.

### Epigenetic effects in affected offspring

Figure 1 summarizes key regions of methylation disturbance at known imprinted loci in patients exposed to maternal *NLRP5* variants. Complete data on all detected regions of methylation disturbance from targeted testing and Illumina Infinium HumanMethylation450 BeadChip array are provided in Supplementary Data 1 and 2, respectively. Only hypomethylation of imprinted loci was observed by targeted testing of MLID patients. However, the methylation array data available for some MLID patients identified additional regions of dysregulation, the majority hypomethylated with a small number of hypermethylated imprinted loci. Hypomethylation was observed at both maternally and paternally imprinted imprinting control regions, including, for example, both the *H19*- and *KCNQ1OT1*-imprinting control centres on chromosome 11p15. Regions of both hypo- and hypermethylation were observed within the GNAS locus, with hypomethylation of the maternally methylated *GNASAS*-imprinting control region (ICR), and hypermethylation of the neighbouring somatic GNASAS differentially methylated region (DMR), methylated on the paternal allele in inverse relation to the hypomethylation of the ICR. The distribution and severity of methylation disturbance varied between patients; and, as expected given the divergent clinical presentations of probands in families 1 and 2, there were also significant differences in the imprinted loci affected despite the shared genetic exposure to *NLRP5* variants.

The amino-acid conservation, pathogenicity and location within the polypeptide for each variant are illustrated (Supplementary Fig. 1 and Supplementary Table 2). The amino acids affected by the variants were well conserved between primates, with progressive loss of conservation of the surrounding amino acids in more phylogenetically distant species. The variants Cys774Arg, Gln785X and Leu947Pro all lie within the ten LRR of NLRP5; LRR domains are thought to function as the ligand sensors of NLRP proteins, such that ligand-binding causes conformational change permissive for nucleotide binding and oligomerization (reviewed in ref. 10). The variants 386dupPro, Gly555Val and Met567Val lie within the NACHT nucleotide-binding domain, which is thought to mediate the nucleotide-dependent oligomerization of NLRP proteins in response to ligand binding. The two variants Met52Val and Glu76Gln lie within the N-terminal effector domain of the protein. *NLRP5* variants with similar damaging potential were identified in The Database of Short Genetic Variation (dbSNP version 138) and are presented in Supplementary Table 3. All of these variants were rare or undetected within dbSNP.

### Clinical and reproductive effects in *NLRP5* families

The most severe clinical and reproductive outcomes were observed in two of the three mothers with biallelic *NLRP5* mutations. The mother of family 1 had biallelic mutations affecting the NACHT and LRR domains, both predicted to be deleterious. She had no healthy offspring, two children with different imprinting disorders (SRS–MLID at 24 years and BWS–MLID at 30 years), and multiple pregnancy losses with three unrelated partners, including one termination for a presumed molar pregnancy. The mother of family 2 had biallelic variants in the LRR domain, one a truncation and the other predicted to be deleterious. She had two healthy offspring, followed by a period of reproductive wastage, a child with BWS–MLID (at the age of 35 years) and a child with non-specific developmental and marked behavioural problems diagnosed as autism with extreme separation anxiety, born when she was 39 years of age. The mother of family 3 had two NLRP5 variants in *cis*, one near and one within the DAPIN domain. The mother had one healthy child before the proband at the age of 34, presenting with BWS–MLID. The offspring of family 4 have previously been described^11^: she was the affected twin in a discordant monozygotic (D-MZ) pair; interestingly, the mutation in the mother was not inherited by either twin. The affected child of family 5 has previously been described^12^; she received a serendipitous diagnosis of MLID, presenting with atypical clinical features of BWS and Prader–Willi syndrome, and it is also remarkable that her heterozygous variant was present in homozygous form in her mother, though her siblings were reportedly healthy. All mothers with *NLRP5* variants were clinically healthy, and none had methylation disturbance at imprinted loci (Supplementary Data 1; further clinical history is presented in Methods).

The median age of mothers affected by *NLRP5* variants, at the birth of affected offspring, was 34.3 years (range 24–35 years), compared with a median age of 30.1 years among other mothers of MLID patients (range 18–40 years; interquartile range 25.4–33.2 years). No offspring of NLRP5-variant mothers were conceived by assisted reproductive technology (ART), compared with 2 of 20 other MLID (where data were available). One of seven offspring of *NLRP5*-variant mothers was a monozygous twin, compared with 6 of 26 other MLID (Supplementary Data 1).

## Discussion

*Nlrp5* (*Mater*) was the first-described maternal-effect gene, with maternal ablation causing developmental arrest at the two-cell stage in mice^9^. In rhesus macaque, *NLRP5* depletion results in arrested zygote development before the 16-cell stage^13^. NLRP5 is a component of the subcortical maternal complex (SCMC) of proteins (KHDC3L, TLE6, OOEP and NLRP5)^14^ essential for developmental progression beyond the first zygotic cell divisions^15^,^16^. The SCMC is polarized to the external subcortex of the cleavage-stage embryo, such that cells of the inner cell mass—destined to contribute to the embryo—contain lower SCMC levels than external cells destined to form extraembryonic structures^15^. While the role of NLRP5 within the SCMC is not established, it is striking that variants predicted to disrupt its ligand-binding and consequent oligomerization appear to disrupt the epigenetic reprogramming and development of the embryo.

The variants described here were located in conserved residues within the DAPIN, NACHT and LRR domains, but were not predicted to cause total loss of function, which may account for their association with both viable and nonviable outcomes. Two other NLRP genes (*NLRP7* and *NLRP2*) and their binding partners are also associated with a spectrum of reproductive wastage and stochastic disturbance of genomic imprinting^4^,^7^,^17^,^18^,^19^, with known mutations of *NLRP7* being enriched within the NACHT and LRR domains^18^,^19^. It remains possible that the incidence of *NLRP5* mutation in our cohort is underestimated, because further variants (including, for example, large rearrangements or copy number changes, or noncoding regulatory variants) may remain to be identified, which may contribute to the affectedness of some of the families described here. Our findings do not exclude the possibility that variants in other genes may contribute to the MLID seen in the families described here. It is also possible that some MLID cases may be caused by compound heterozygosity of variants in *NLRP5* and its functional partners. Further exome analysis of patients and mothers is ongoing to address these questions. In addition, functional analysis is required to determine how *NLRP5* coding variants alter the interactions between NLRP5 and its binding partners, and whether these reduce the developmental competence of the oocyte, or compromise its intrinsic biology or stability, or deplete the oocyte pool in affected females. Moreover, given the existence of other rare but potentially deleterious missense and nonsense variants within its coding sequence (Supplementary Table 3), *NLRP5* warrants investigation as a genetic factor in female reproductive problems.

The imprinting disturbance in offspring of mothers with *NLRP5* variants comprised variable loss of DNA methylation at both maternally and paternally imprinted germline imprints in different individuals, suggesting that the maternal effect of the mutation is exerted in the first cell divisions of the fertilized zygote. It may be that the variants caused much broader epigenetic disturbance in the concepti of affected mothers, but the natural epigenetic resetting of development^1^ corrected most disturbances, leaving only imprinting disturbances detectable postnatally.

The phenotypes of affected offspring were variable, with growth and neurodevelopmental problems. While the majority of probands had clinically recognizable imprinting disorders, two of seven cases had clinical features not consistent with a diagnosis of any imprinting disorder. Therefore, it is likely that in general, such patients remain undiagnosed. Although patient numbers in this study are too low to describe trends, the severity of clinical outcome seemed to reflect both the severity of *NLRP5* mutation and the age of the mothers at the birth of their offspring, in that four of five mothers had healthy offspring before the offspring were affected by MLID. We observed that affected mothers with NLRP5 variants had a median age of 34.1 years, as compared with other MLID mothers (30.1 years) and the median maternal age for all births in the general population 29.5 years (http://www.ons.gov.uk/ons/rel/vsob1/birth-statistics--england-and-wales--series-fm1-/no--29--2000/index.html). It is well recognised that the progressive decline in female reproductive fitness may reflect non-genetic factors such as the chronological age of the mother, or the reproductive age of the oocyte. Dankert *et al*.^20^ recently reported that *in vitro* aged oocytes show a decline in the abundance of transcripts including *NLRP5*, suggesting that delay in fertilization may jeopardize oocyte fitness.

The affected offspring of mothers with NLRP5 variants included a D-MZ twin pair. Some imprinting disorders are associated with an elevated rate of twinning, almost invariably monozygotic and discordant, and enriched for MLID, but the causal relationship between imprinting disorders and twinning has remained unclear^21^. Notably, among MLID patients without NLRP5 involvement, 6 of 26 (23%) were D-MZ twins, far in excess of the ∼3% in the general population. There is also a recognized excess risk of imprinting disorders in children conceived by ART though the absolute risk of imprinting disorder in ART remains low^22^. None of the children affected by MLID with *NLRP5* involvement were conceived by ART, though one sibling (family 4) was conceived by ART, which may be indicative of reproductive difficulties. By comparison, 2 of 20 other children in the MLID cohort were conceived by ART, against a population rate of ∼2% (http://www.hfea.gov.uk/docs/HFEA_Fertility_Trends_and_Figures_2013.pdf). The relationship between imprinting disorders, ART and age of parents is complex. ART is often sought by parents with fertility problems, and it may be that the risk of imprinting disorders is associated less with ART *per se* than with the reduced fertility of the parents or the increase in age following a delay in conception. The small case numbers in this study and the potential for ascertainment bias preclude definitive conclusions, but our observations suggest that collection of detailed data on ART, twinning, fertility history and parental ages in families affected by MLID may reveal some novel associations.

The finding of *NLRP5* mutations in five mothers of offspring with MLID suggests links between maternal-effect genes, maternal reproductive fitness, epigenetic and developmental reprogramming of zygotes, and reproductive outcomes. We suggest that *NLRP5* mutations may have been overlooked in the past as a cause of imprinting disorders or reproductive problems because their effects encompass both, and might not be captured by studies of either alone; an integrated investigation of the relationship between NLRP5, imprinting disorders and reproductive wastage is now warranted. Furthermore, maternal effect genes in general may be underestimated as a cause of developmental problems in children. Recent technological advances in the epigenetic analysis of gametes and zygotes^23^,^24^ now make it possible to explore the impact of genetic mutations and environmental insults on developmental and epigenetic reprogramming in early development.

## Methods

### Ethics

All patients were consented into the research study ‘Imprinting disorders—finding out why’ (IDFOW: Southampton and South West Hampshire Research Ethics approval 07/H0502/85) through the UK Comprehensive Local Research network (www.southampton.ac.uk/geneticimprinting/informationpatients/imprintingfindingoutwhy.page, accessed on September 2013), with the exception of the patient in family 4 and patients 17–23 who were consented into the research study ‘Disorders caused by imprinting defects’ funded by the Bundesministerium für Bildung und Forschung (BMBF grant 01GM1513), and approved by the Ethical committee of the University Hospital Aachen, Germany.

Because the consent framework of the IDFOW study does not encompass the deposition of whole-exome sequence data in an open-access repository, whole-exome sequence files are instead deposited in a restricted-access repository under doi:10.5258/SOTON/378548. Data access are managed by the Wessex Imprinting Governance Team, to which application can be made by contacting the Corresponding Author, or the Study Administrator (via www.southampton.ac.uk/geneticimprinting/informationpatients/imprintingfindingoutwhy.page).

### Whole-exome sequencing

Whole-exome sequencing for samples from the IDFOW cohort was performed on DNA derived from peripheral blood, and sequenced on an Illumina HiSeq2000 sequencer at the Wellcome Trust Centre for Human Genomics using the Agilent SureSelect v5 capture kit encompassing 51 Mb of genome sequence. Exome sequencing of the parents and proband of family 4 and four additional SRS–MLID families was performed using the NimbleGen Human SeqCap EZ v3.0 Kit and the Illumina HiSeq2000 system for sequencing according to the manufacturers’ protocols. Paired-end exome sequence reads were aligned to the hg19 human reference genome using Burrows–Wheeler Aligner (BWA-MEM v 0.7.5a) to produce binary sequence alignment format (BAM) files and Picard (v1.95) was used to remove duplicate reads. Local indel realignment and base quality recalibration were performed using the Genome Analysis Toolkit (GATK v3.0–0) before the realigned and recalibrated BAM files were used to determine single-nucleotide variants including SNPs and indel (insertion–deletion) alleles. GATK was used to predict and genotype variants for each sample, raw variant calls were outputted in variant call format file, and variant filtration was performed for both SNPs and indels to remove low quality and potentially false-positive variants. Variant data were annotated using Annovar (v 2013Aug23) and KggSeq (v 0.6).

### Sanger sequencing

Sanger sequencing of *NLRP5* was used to confirm exome variants, establish their inheritance, fill gaps in exome coverage and screen a further 14 patients (four BWS–MLID, five SRS–MLID, three transient neonatal diabetes mellitus–MLID and two idiopathic-MLID) and 19 mothers of individuals with MLID.

M13 universal tagged primers were designed to 14 of the 15 exons of NLRP5 (see Supplementary Table 4). Exons were amplified using Q5 High-Fidelity DNA Polymerase (New England BioLabs). Amplicons were then treated with *ExoSAP* to degrade any remaining primers, before sequencing with M13 forward and reverse primers using BigDye 1.1chemistry (Applied Biosytems). Sequencing reactions were analysed on an ABI Prism 3130XL sequencer (Applied Biosystems).

Co-amplification of exons 5 and 6 was observed with both primer sets due to their highly similar sequence and required the use of internal sequencing primers with exon-specific terminal-3′ bases (see Supplementary Table 4) to generate exon-specific sequencing. Repetitive sequence at Exon 4 required the use of primers (see Supplementary Table 4) without M13 universal tags and Phusion High-Fidelity DNA Polymerase (New England BioLabs) to generate the sequencing template. Exon 4 amplicons were sequenced as previously described using the amplification primers.

### *In silico* prediction of variant pathogenicity

The pathogenicity (SIFT, Polyphen2 and PROVEAN) scores of the variants identified was predicted using the online tools Ensembl Variant Effect Predictor (http://www.ensembl.org/Homo_sapiens/Tools/VEP), Polyphen-2 (http://genetics.bwh.harvard.edu/pph2/) and PROVEAN v1.1.3 (http://provean.jcvi.org/index.php), applied with standard procedures and settings and presented in Supplementary Table 2. These tools were also used to predict the pathogenicity of *NLRP5* variants in dbSNP138. Nonsense variants and variants predicted to be pathogenic by one or more programmes are presented in Supplementary Table 3.

### Epigenetic and epigenomic analysis

For patients from the IDFOW cohort and mothers of families 1–5, targeted methylation-specific PCR (MSP) analysis was performed on bisulfite-converted DNA (Zymo Research, Orange, CA). MSP was used to determine DNA methylation at 17 DMRs of imprinted genes^25^. For each DMR, bisulfite treated DNA was competitively amplified using forward primers derived from methylated or unmethylated genomic DNA, against a common fluorescently labelled reverse primer (Supplementary Table 5) using HotStar DNA polymerase (Qiagen, Hilden, Germany). Products amplified in a ratio reflecting that of genomic source DNA were visualized by capillary electrophoresis on an ABI 3130 Genetic Analyzer (Applied Biosystems, Foster city, CA), and then peak height ratiometry was calculated and normalized to control samples. NESP DNA methylation was determined using the SALSA MLPA ME031 probemix, MRC-Holland, according to the manufacturer’s instructions. DNA methylation analysis in patients from the BMBF cohort used methylation-specific single-nucleotide primer-extension (MS-SNuPE) of eight loci and methylation-specific multiplex ligation-dependent probe amplification (MS-MLPA) analysis (SALSA MLPA ME030 BWS/RSS probemix, MRC-Holland) of a further two loci. For MS-SNuPE analysis a primer was designed to end directly in front of the C of each CpG of interest. After bisulfite conversion (Zymo Research, Orange, CA) and amplifying PCR, this primer is elongated by one base. The incorporated base corresponds to the methylation status of the CpG. This information can be used to calculate the degree of differential methylation at the CpG. For each sample, bisulfate-treated DNA was amplified in a multiplex PCR using the QIAGEN Multiplex PCR kit (Qiagen, Hilden, Germany) with the SNuPE-PCR primer mix (Supplementary Table 6). Excess primers were degraded using ExoSAP (USB, Cleveland, OH, USA) before the primer-elongation reaction with ABI Prism SNaPshot Multiplex Ready Reaction Mix (Life Technologies, Darmstadt, Germany) and either SNuPE-Primer-Mix 1 or 2 (Supplementary Table 6) according to the manufacturer’s protocol. The final product was resolved on an ABI 3130 Genetic Analyzer (Applied Biosystems, Foster city, CA) and analysed using GeneMapper Software 4.0 (Life Technologies, Darmstadt, Germany). Peak areas were used to calculate percentage of methylation. In each assay 3–4 normal controls were analysed and used for normalization^26^.

Genome-wide DNA methylation analysis was generated using the Illumina Infinium HumanMethylation450 BeadChip (Illumina, Inc., CA, USA) and a single-sample analysis pipeline^27^. Significant deviation of methylation from a group of 50 normal controls was defined as a *P* value (adjusted using false discovery rate) <0.05 across a minimum of three consecutive CpGs within 2,000 nucleotides; the additional requirement of *M* values between −1 and +1 in normal controls was applied to enrich differential methylation at loci consistent with genomic imprinting. Regions associated with known imprinted genes (http://igc.otago.ac.nz/home.html) or loci previously identified as disregulated in individuals with MLID^28^ are presented for probands from families 1, 2, 3 and 5 in Supplementary Data 2.

### Copy number variation

For digital PCR, genomic DNAs were fragmented by MseI digestion (New England Biolabs) for five normal controls and one sample where a novel homozygous variant was identified in a region of homozygosity by Sanger sequencing. Droplet digital PCR amplification of two regions of exon 7 generated a 91- and 90-bp amplicon, respectively, in 20-μl reactions containing 64-ng digested DNA, using 900 nM primers (NLRP5 ex7 CNV 1F and NLRP5 ex7 CNV 1R or NLRP5 ex7 CNV 2F and NLRP5 ex7 CNV 2R, respectively, Supplementary Table 1), 200-nM probe (5′FAM, 3′BHQ1; NLRP5 ex7 CNV 1probe or NLRP5 ex7 CNV 2probe, Supplementary Table 1), 1 × PrimePCR KRAS wild-type for pG12D (human) mutation assay primer/probe mix (Bio-Rad Laboratories Inc., Hercules, CA) and 1 × droplet digital pcr supermix for Probes (no dUTP; Bio-Rad Laboratories Inc.).

Droplets were generated using the QX200 Droplet Generator (Bio-Rad Laboratories Inc.) and 70 μl Droplet Generation Oil (Bio-Rad Laboratories Inc.). Droplet PCR cycling conditions were 95 °C for 10 min, then 40 cycles of 94 °C for 30 s, 55 °C for 60 s and final enzyme deactivation at 98 °C for 10 min. Fluorescent droplets containing amplified products were read using the QX200 Droplet Reader (Bio-Rad Laboratories Inc.). Copy number variation of *NLRP5* relative to the wild-type *KRAS* reference was calculated by the formula (concentration of *NLRP5*/concentration of *KRAS*) × copy number of *KRAS* reference (=2).

### Patients

In family 1, patient 1 (atypical SRS), was born at 32 weeks gestation with a birth weight of 1,040 g (<0.4th centile). She had a cleft palate, a right unilateral cleft lip and bilateral fifth finger clinodactyly. There were feeding difficulties for the first 6 months of life requiring nasogastric tube feeds. By two years of age minimal body asymmetry was documented (with the left side appearing smaller than the right); her height and weight were on the 0.4th centile with a head circumference on the 9th centile. A diagnosis of SRS was made and confirmed using routine NHS testing for hypomethylation at H19. In childhood, she showed mild developmental delay and behavioural difficulties including poor sleep and self harm. By 9 years of age her height, weight and head circumference were all on the 25th centile without treatment, and the asymmetry was less marked. There were ongoing concerns regarding her behaviour and she required significant additional help in a mainstream primary school, including a statement of educational need and, speech and language therapy. Her mother reported that throughout her childhood, patient 1 showed episodic excess sweating, but there have been no documented episodes of hypoglycaemia. Investigations including calcium, phosphate and parathyroid hormone were normal.

Patient 2 in family 1 (BWS), the maternal half-brother of patient 1, was born at 36 weeks gestation with a birth weight of 3,425 g (between the 91st and 98th centiles), and a head circumference of 33.7 cms (50th centile). During the pregnancy, antenatal ultrasound scans identified a cystic appearance of the placenta and by 30 weeks, macroglossia was diagnosed. Post-delivery maternal β-HCG levels were normal. At birth macroglossia was confirmed and he was also reported to have bilateral ear creases and a natal tooth. During the neonatal period there was one documented episode of hypoglycaemia. By the age of three months, Patient 2 had developed an umbilical hernia. He was diagnosed with BWS and this was confirmed on routine NHS testing to show a loss of methylation at ICR2. There was no hemihypertrophy. At around 1 year of age a renal ultrasound scan revealed discrepant size of the kidneys, but no evidence of a tumour.

There was no further family history of SRS, BWS or any other imprinting disorder; however, the mother of these two children had suffered significant pregnancy related morbidity. She had six pregnancy losses, with three unrelated partners, and one medical termination of pregnancy for a presumed molar pregnancy, associated with a developing fetus (interpreted as either a partial mole or a twin pregnancy with one twin developing as a hydatidiform mole). Aside from the pregnancy losses, there was no other medical history of note: she herself was born with a normal birth weight to non-consanguineous parents. Her own mother had suffered three miscarriages.

In family 2, patient 1, (BWS) was the third of four children, born when the mother was 35 years of age. He was born at 32 weeks of gestation with a birth weight of 1,700 g, following a diagnosis of preeclampsia in his mother. The placenta was described as showing mesenchymal dysplasia. He had an uneventful neonatal period, requiring tube feeding initially but not ventilation, and was discharged at 4 weeks of age. He was admitted following an episode of apnoea at 3 months, and at that time was diagnosed with macroglossia, though surgical resection was not performed. He was also noted to have an umbilical hernia, undescended testes and hemihypertrophy. A diagnosis of BWS was made and confirmed on routine NHS testing to show hypomethylation at ICR2. He had an episode of pneumonia at 5 months and was diagnosed with asthma. He had delayed development, walking at 18 months and was diagnosed with speech delay and subsequently dyslexia and dyscalculia. He required an educational statement and is currently educated at a special school. On examination at 8 years 5 months, his height was 126.5 cm (25th centile), weight 28.9 kg (50th–75th centile) and occipital frontal circumference (OFC) 54 cm (90^th^ centile) and by 13 years his height was 161.7 cm (75th centile) and weight 55 kg (90th centile). He had a naevus flammeus on his forehead. He was noted to have asymmetry, with the right side of the face, tongue and right leg being larger than the left (2-cm difference in foot length), and showed a mild positional scoliosis.

Patient 2 in family 2 was the fourth of four children, born when mother was 39 years of age. Pregnancy was normal with a birth weight of 3,118 g at term. He fed well, but made a rapid gain in weight. Obesity was diagnosed at 16 weeks, when he weighed 9.22 kg (>99.6th centile). At 11 months he weighed 14.2 kg and a diagnosis of BWS was considered because of excessive weight gain and a forehead naevus flammeus; but routine testing was negative. He walked at 17 months and started to say single words at 2 years, but did not progress to speaking in sentences until 4 years, and marked expressive speech delay was diagnosed. He required special education, with an educational statement of need. At the age of 8 years and 6 months, he had markedly unusual behaviour, with extreme separation anxiety such that he was able to attend school only on three mornings a week. He was diagnosed with autism, with episodes of severe anger. He was constantly hungry, but did report satiety after eating, and there was no history of stealing food. He suffered from gastrointestinal reflux and severe constipation. His general health was good. On examination aged 8 years and 6 months his OFC was 58 cm (98–99.6th centile), his height at 134.8 cm was on the 75th centile, and his weight was 54.4 kg well above the 99.6th centile. He had a round face with long, narrow palpebral fissures, a short nose with anteverted nares, normal ears and tongue, no clinical asymmetry. He had hyperextensible fingers but no evidence of shortening of the metacarpals. He was prepubertal. The rest of the examination was normal.

There were two older siblings with normal development and growth (male and female) born 3 years and 2 years before patient 1. The older male sibling had mild anxiety in early childhood but is doing well at school with well above-average educational attainment. Mother suffered one miscarriage before patient 1 and 3 miscarriages before patient 2.

The proband in family 3 was the son of young, healthy and unrelated parents. He was born at 39 weeks gestation with a birth weight of 3,500 g (50th centile), birth length of 53 cm (90–97th centile) and a head circumference of 36 cm (50th–90th centile). At birth he was confirmed to have macroglossia, cheek and tongue right-side hemihyperplasia, a naevus flammeus of the forehead and occipital region. A diagnosis of BWS was made but no episodes of hypoglycaemia were reported during the neonatal period. At around 2 years, diastasis recti was observed, but surgery was not needed. At the age of two and a half years the child had age-appropriate psychomotor development and abdominal scanning was normal. He has one healthy elder brother, and no miscarriages were documented.

The proband in family 4 has previously been published as patient 3 (ref. 11). The female proband presented neonatally and was a monozygotic twin; her sister is healthy. After a pregnancy reported as normal, the twins were born at 31 weeks of gestational by Caesarean section. The affected twin’s birth weight was 995 g (−1.88 s.d.), length 35 cm (−1.74 s.d.) and head circumference 28 cm (−0.45 s.d.). She was diagnosed with SRS because of growth restriction, relative macrocephaly, facial gestalt (prominent forehead, triangular face, downturned corners of the mouth, micrognathia), asymmetry and clinodactyly of the fifth digit. During the first days of life, gastric tube feeding was required. Her twin sister showed birth measurements within the normal range.

The family history was unremarkable. The German parents were not consanguineous; the maternal age at birth was 30 years and paternal age was 33 years. The twins were the product of a normal conception, but their elder (healthy) sibling was the product of assisted reproductive therapy and has normal growth and psychomotor development.

The proband in family 5 has previously been reported^12^. The proband is the third of four children, born to healthy Tamil parents with no reported consanguinity; the mother was aged 34 and the father 42 years at her birth. After an unremarkable pregnancy she was born at 42 weeks’ gestation with a birth weight of 3,460 g. Her neonatal course was unremarkable; she fed well, though macroglossia was noted. On examination aged 3 years 6 months her height was 97 cm (25–50th centile) and weight 17 kg (75–91st centile). She had persistent tongue protrusion, mild facial dysmorphism, mild hypotonia, speech and language difficulties, social communication problems and extreme shyness. Her unusual clinical features prompted molecular genetic testing for both BWS and Prader–Willi syndromes, but she was found to have mosaic imprinting disturbance at multiple loci.

No details of health or reproductive issues are reported among other family members, except for atrial septal defect and ventral septal defect in one sibling.

## Additional information

**Accession codes:** Exome sequence data for the patients in families 1–4 have been deposited in http://eprints.soton.ac.uk/id/eprint/378548 under the accession codes 378548.

**How to cite this article:** Docherty, L. E. *et al*. Mutations in *NLRP5* are associated with reproductive wastage and multilocus imprinting disorders in humans. *Nat. Commun.* 6:8086 doi: 10.1038/ncomms9086 (2015).

## Supplementary Material

Supplementary Figure and Tables Supplementary Figure 1 and Supplementary Tables 1-6

Supplementary Data 1 Targeted DNA methylation testing

Supplementary Data 2 Imprinted loci identified by Illumina Infinium Human450 methylation array
