# Supplementary material for: Mutations in NLRP5 are associated with reproductive wastage and multilocus imprinting disorders in humans
Source: Nat Commun. 2015 Sep 1;6:8086. doi: 10.1038/ncomms9086 (PMC4568303; doi:10.1038/ncomms9086)
Supplement: Supplementary Figure and Tables — Supplementary Figure 1 and Supplementary Tables 1-6 [file ncomms9086-s1.pdf]

Supplementary Figure 1: NLRP5 Variant amino acid conservation and pathogenicity

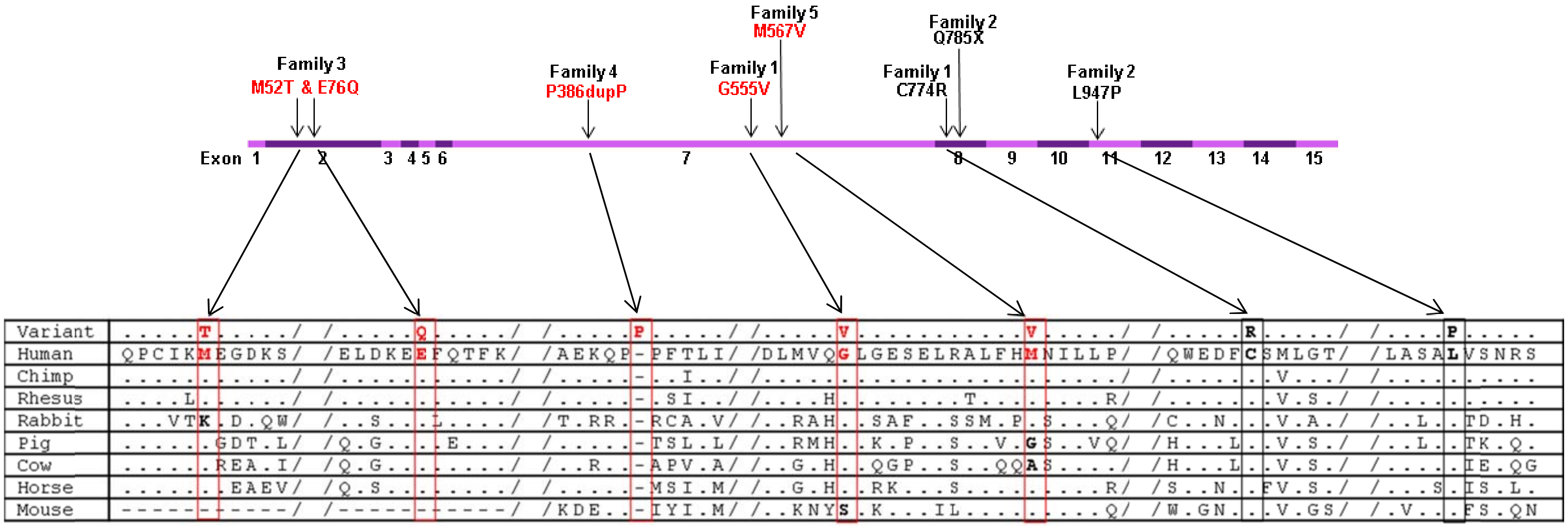

Diagrammatic structure of human *NLRP5* cDNA with variant positions shown and expanded below to show the conservation of the amino acid affected variant (boxed amino acid) and five flanking amino acids across several species (novel variants are highlighted by red text). The human single letter amino acid reference sequence is provided with separation between regions indicated (/ /). Where amino acid sequence is not conserved the single letter amino acid code specific for that species is provided with conserved amino acids and gaps in alignment indicted by . and – respectively.

Supplementary Table 1: Copy Number Variation analysis of NLRP5.

Sample	Control primers (HEX)	Target primers (FAM)	Copy number
Family 5 Mother	KRAS WT pG12D (Bio-RAD)	**NLRP5 ex7 CNV 1F** CACGGAAAGTCAGGAATTGG<br>**NLRP5 ex7 CNV 1probe** CCAGAAGGATCGTGCTGTGCTGGGCG<br>**NLRP5 ex7 CNV 1R** AGGAGAACATTCCCTGGTAG	1.97
Family 5 Mother	KRAS WT pG12D(Bio-RAD)	**NLRP5 ex7 CNV 2F** AAGCTTCTGCACTGGGTC<br>**NLRP5 ex7 CNV 2probe** CAGCCTAATGCCACCACCCCAGGAG<br>**NLRP5 ex7 CNV 2R** CTCGAAAAGACAGTGGAAGG	2.04

Digital PCR results of two regions of NLRP5 exon 7 compared to a KRAS control for pedigree 5 mother.

Supplementary Table 2: Pathogenicity of NLRP5 variants in families 1-5.

Variant	Polyphen		SIFT		PROVEAN		Family	dbSNP or novel
	Score	Status	Score	Status	Score	Status		
Gly555Val	1	Probably damaging	0.014	Damaging	-6.72	Deleterious	1	Novel
Cys774Arg	0.992	Probably damaging	0.003	Damaging	-9.71	Deleterious	1	rs370837790
Gln785X	N/A	Not analysed	N/A	Not analysed	-10.5	Deleterious	2	rs200446614
Leu947Pro	1	Probably damaging	0.001	Damaging	-6.27	Deleterious	2	rs202181446
Met52Thr	0.075	Benign	0.003	Damaging	-1.4	Neutral	3	Novel
Glu76Gln	1	Probably damaging	0	Damaging	-1.65	Neutral	3	Novel
386insPro	N/A	Not analysed	N/A	Not analysed	-5.56	Deleterious	4	Novel
Met567Val	0.032	Benign	0.37	Damaging	-1.78	Neutral	5	Novel

Polyphen 2 scores range from 0 (neutral) to 1 (damaging), Sift scores range between 0 and 1 with scores <= 0.05 and > 0.05 defined as damaging and tolerated respectively and Provean scores <-2.28 defined as deleterious.

Supplementary Table 3: **Deleterious NLRP5 dbSNP variants.**

dbSNP	Minor allele frequency	Variant	Polyphen2		SIFT		PROVEAN	
			Score	Status	Score	Status	Score	Prediction
rs143801460	0.0045	Tyr70Cys	0.455	possibly_damaging	0.01	deleterious	-1.42	Neutral
rs199871361	0	Glu100Asp	0.952	probably_damaging	0.09	tolerated	-1.67	Neutral
rs201123213	0	Arg143Trp	0.994	probably_damaging	0	deleterious	-3.49	Deleterious
rs45627733	0.0008	Thr281Met	1	probably_damaging	0	deleterious	0.004	Damaging
rs149735408	0	Gln305Arg	0.046	benign	0.38	tolerated	0.007	Damaging
rs189035436	0	Val316Ile	0.209	benign	0.2	tolerated	0.002	Damaging
rs201835640	0	Glu339Lys	0.715	possibly_damaging	0.01	deleterious	0.021	Damaging
rs200541204	0	Arg356Gly	0.988	probably_damaging	0	deleterious	0.172	Tolerated
rs191551009	0	His508Asp	0.965	probably_damaging	0.01	deleterious	0.228	Tolerated
rs200560950	0.0023	Cys519Arg	0.081	benign	0.1	tolerated	0.01	Damaging
rs34175666	0	Arg562His	0.54	possibly_damaging	0.02	deleterious	0.045	Damaging
rs201845628	0	Leu571Phe	0.828	possibly_damaging	0.04	deleterious	0.177	Tolerated
rs35696923	0.0106	Ser586Arg	0.994	probably_damaging	0	deleterious	0.197	Tolerated
rs200704861	0	Ser586Thr	0.989	probably_damaging	0.01	deleterious	0.694	Tolerated
rs200446614	N/A	Gln785X	0.794	possibly_damaging	N/A	Not analysed	N/A	Not analysed
rs200340956	0	Thr794Arg	0.093	benign	0.01	deleterious	0.019	Damaging
rs144876069	0.0008	Arg796Trp	0	benign	0.59	tolerated	0.046	Damaging
rs182774527	0.0053	Arg837Cys	0.026	benign	0.05	tolerated	0.005	Damaging
rs201517651	0	Arg837His	0.018	benign	0.35	tolerated	0.043	Damaging
rs199475780	0.0045	Cys858Arg	0.87	possibly_damaging	0.06	tolerated	0.032	Damaging
rs200240705	N/A	Glu869X	0.015	benign	N/A	Not analysed	N/A	Not analysed
rs199886442	0	Asp908His	0.973	probably_damaging	0.01	deleterious	0.017	Damaging
rs192219590	0.0008	Gly910Glu	0.811	possibly_damaging	0.02	deleterious	0.002	Damaging
rs34394321	N/A	Leu943ThrfsTer39	0	benign	N/A	Not analysed	N/A	Not analysed
rs141696788	0	Asn965Lys	0.008	benign	0.43	tolerated	0.037	Damaging
rs182963698	0.0076	Ala1003Val	0.151	benign	0.03	deleterious	0.176	Tolerated
rs367661215	0	Leu1057Val	0.388	benign	0.02	deleterious	0	Damaging
rs201338049	0	Thr1073Met	0.823	possibly_damaging	0.04	deleterious	0.062	Tolerated
rs377588935	0	Ala1097Val	0.001	benign	0.04	deleterious	0.056	Tolerated
rs149176940	0	Cys1104Gly	0.999	probably_damaging	0.01	deleterious	0	Damaging
rs12462795	0.0726	Ser1108Cys	0.976	probably_damaging	0	deleterious	0.009	Damaging
rs142260362	0	Ser1126Ile	0.994	probably_damaging	0	deleterious	0.03	Damaging
rs200444348	0	Leu1142Val	0.999	probably_damaging	0	deleterious	0	Damaging
rs201631280	N/A	Arg1180X	0.104	benign	N/A	Not analysed	NA	Not analysed

A summary of NLRP5 dbSNP138 stop variants and variants predicted to be pathogenic by Polyphen 2, SIFT or PROVEAN analysis. Polyphen 2 scores range from 0 (neutral) to 1 (damaging), SIFT scores range between 0 and 1 with scores <= 0.05 and > 0.05 defined as damaging and tolerated respectively and PROVEAN scores <-2.28. The minor allele frequency annotated by dbSNP138 has been included (a score of 0 indicates that it has not been observed in the dbSNP138 population and N/A where no frequency data were available) along with variant information.

Supplementary Table 4. NLRP5 Sanger sequencing primers.

Primer name	Sequence
NLRP5 Ex1 F M13	TGTAAAACGACGGCCAGTATACTCTTCTGCTCAGGACGTCT
NLRP5 Ex1 R M13	CAGGAAACAGCTATGACCCCTGGACAACTGTCATGGGTTGA
NLRP5 Ex 2 F M13	TGTAAAACGACGGCCAGTATGTCCCAGCACTGATGTCT
NLRP5 Ex 2 R M13	CAGGAAACAGCTATGACCACGATTCTTCCCTGTTCCCA
NLRP5 ex 3 F M13	TGTAAAACGACGGCCAGTGGCCAATCATGGAATAAACTGAGAT
NLRP5 ex 3 R M13	CAGGAAACAGCTATGACCCCACAGAACTCTTCAACTTGGAAA
NLRP5 ex 4 seq F	AGCCAGTGGTTCTGTGCTCT
NLRP5 ex 4 seq R	ATTTCCCCGTCCTTTGAACT
NLRP5 ex 5 F M13	TGTAAAACGACGGCCAGTCAAGGCTCTTCAAGGAGAAATGAG
NLRP5 ex 5 seq F	TCTAGCTGAGACAGTGGTTGC
NLRP5 ex 5 R M13	CAGGAAACAGCTATGACCGGTTGACACCAGAGACAAATGAGAA
NLRP5 ex 5 seq R	CACCAGAGACAAATGAGAA
NLRP5 ex 6 F M13	TGTAAAACGACGGCCAGTAGTGCACTCTGTCTTCTAGTTGAGC
NLRP5 ex 6 seq F	AGTTGAGCCGGTGGTTGT
NLRP5 ex 6 R M13	CAGGAAACAGCTATGACCTTGGCCATATGTTTAACATCCATCA
NLRP5 ex 6 seq R	CACCAGAGACAAATGAGAT
NLRP5 ex 7 F1 M13	TGTAAAACGACGGCCAGTTGGCTTCAAATTTTGTTTCCCGATT
NLRP5 ex 7 R1 M13	CAGGAAACAGCTATGACCGAAGCCCCACCGGTCTGAAT
NLRP5 ex 7 F2 M13	TGTAAAACGACGGCCAGTTGGCTTCAAATTCGCTGAGGAGGAGGATGTA
NLRP5 ex 7 R2 M13	CAGGAAACAGCTATGACCTGACGGTGACGATCAGGAAGGA
NLRP5 ex 7 F3 M13	TGTAAAACGACGGCCAGTTGGCTTCTCTGCAAAGACTGGGCTGAGAAG
NLRP5 ex 7 R3 M13	CAGGAAACAGCTATGACCAAACACTGACTTCCTATTCCACACT
NLRP5 ex 7 F4 M13	TGTAAAACGACGGCCAGTTGGCTTCATCAGCTCACCCCTCGAGGC
NLRP5 ex 7 R4 M13	CAGGAAACAGCTATGACCCAACAGAGAGACCCAGTGCAGAA
NLRP5 ex 7 F5 M13	TGTAAAACGACGGCCAGTCCTCGTGAGCGAAGACGTAAG
NLRP5 ex 7 R5 M13	CAGGAAACAGCTATGACCCATTTTAGCCAGCTGGACGTCAT
NLRP5 ex 8 F M13	TGTAAAACGACGGCCAGTTCCCTCTCCTCCGACGTGTTG
NLRP5 ex 8 R M13	CAGGAAACAGCTATGACCCTTTAGCGACTCCCCACTCTGT
NLRP5 ex 9 F M13	TGTAAAACGACGGCCAGTGACACCAGGTTTGGACGGGTTT
NLRP5 ex 9 R M13	CAGGAAACAGCTATGACCTGCAGCTCTTTAATTTTTGCCACTT
NLRP5 ex 10 F M13	TGTAAAACGACGGCCAGTGAAATGGGCTGCTTTGGACA
NLRP5 ex 10 R M13	CAGGAAACAGCTATGACCGGTCCCAACTTCCTTCCTCA
NLRP5 ex 11 F M13	TGTAAAACGACGGCCAGTGGATGCGACTTCACCATATGTC
NLRP5 ex 11 R M13	CAGGAAACAGCTATGACCTTCCCACTGTAAGATAACACAAAGG
NLRP5 ex 12 F M13	TGTAAAACGACGGCCAGTGAGGAGAGCAGCAGCTCACTTGA
NLRP5 ex 12 R M13	CAGGAAACAGCTATGACCGGAATGCACAGTGTTTAGGTATCAAGG
NLRP5 ex 13 F M13	TGTAAAACGACGGCCAGTAACTCCAGTTAATGCTGCTGAACCT
NLRP5 ex 13 R M13	CAGGAAACAGCTATGACCCAATCGGCAGTTGAGGGTTTTCTAC
NLRP5 ex 14 F M13	TGTAAAACGACGGCCAGTCAAGGTGAAATTCATACGGGTTGA
NLRP5 ex 14 R M13	CAGGAAACAGCTATGACCTAAAATGTGGCTCCCAAGGTATGA
NLRP5 ex 15 F M13	TGTAAAACGACGGCCAGTATGAGGCTACCAGGAATTTTGAAGA
NLRP5 ex 15 R M13	CAGGAAACAGCTATGACCACCACTTGACAACTTTTCCAAATGC

M13 universal tagged primers designed to 14 of the 15 exons of NLRP5 with untagged primers for exon 4 and internal sequencing primers for exons 5 and 6

Supplementary Table 5: Methylation-specific PCR primers.

DMR	Chromosomal location hg19	Methylated allele size	Unmethylated allele size	Methylated primer	Unmethylated primer	Universal FAM labelled primer
DIRAS3	chr1:68516329-68516510	mat 182	pat 167	CGCGGTTTTATATTTCGATTTGCGTATAGGAGCG	TGATTTGTGTATAGGAGTGGGGTGTGGGGGTG	CTTCTAAACTAACCCCTCRATTATTATAAATACC
PPIEL	chr1:40025245-40025376	mat 120	pat 126	CGGTGCGGGTTTTCGGCGGAAGC	TGGGGTATGGTGTGGGTTTTTGGTGGAAGT	CACCCCCAACTCAATCTTAACACTACCTAC
PLAGL1	chr6:144329285-144329476	mat 175	pat 187	TTCGGGGAAGCGTTTCGCGCGTTAAGGTT	GGTTATGATGGTGATTTGGGGAAGTGTTTTG	CACRACATCTACCATTTATCATTCAACC
IGF2R	chr6:160426774-160427044	mat 256	pat 270	GAGGAGGCGAGGGGCGCGGCGAGGCGGC	GTGTGGAGGGGTATGAGGAGGTGAGGGGTGTGGTG	CCATTCTCTCCCCTCCCCAACTACAACCTTCC
GRB10	chr7:50849940-50850136	mat 184	pat 190	CGGTAGGCGGGTAGGGGGTCGCGC	GTGAGTTTGTGGTAGGTGGGTAGGGGGTTGTGTG	CCRCCCRCTCTCCAAATACTCAAATAAACTCC
MEST	chr7:130132121-130132215	mat 211	pat 199	CGGAGTGGTTGTAGTTGTTCGGCGCGGC	GTAGTTGTTTGGTGTGGTGTTGTTTTGTGTGGG	CCAACCACACCCCCCTCRTTCCCACC
H19	chr11:2019455-2019764	pat 295	mat 305	CGTTTGTTAGTAGAGTGCGTTCGCGAGTCG	GGTTGTTTATTGTTTGTTAGTAGAGTGTGTTTGTG	ATAACAGAAAAAACCCCTTCCTACCACCATCAC
IGF2 DMR0	chr11:2169485-2169651	pat 155	mat 163	GTTTGACGAGGTTAGTGAGGGACGGCG	ATAGTTTTGTTTGAtGAGGTTAGTGAGGGATGGTG	CCAAAACAATTTCCCTAAAAATACTCATTCATAC
KCNQ1OT1	chr11:2721195-2721469	mat 270	pat 210	TTCGTGTTGAGGCGACGCGGCGATCGTTTTGTT	TTTGTTGTTGTTGATGTGGTGATTGTTTTT	CCACCTCACACCCAACCAATACCTCATA
MEG3	chr14:101292454-101292679	pat 226	mat 199	CGCGTTTTGGTTCGTTGGTTTTGGCGGCG	GtGTAGAtGGtGGAGAGTAGAGAGGGAGtGtG	CTCCAACAACAAAACCCAAAATCAAACAAACTCTC
SNRPN	chr15:25200031-25200342	mat 310	pat 218	TATTGCGGTAAATAAGTACGTTTGCGCGGTC	GTGAGTTTGGTGTAGAGTGGAGTGGTTGTTG	CTCCAAAACAAAAAACTTTAAAACCCAAATTCC
IGF1R	chr15:99409197-99409370	mat 171	pat 178	ACCGAATTCAACTAAAAACGCCCGCACG	CAATAAAACCAAATTCAACTAAAAACACCCACAC	GTAGGTAGGAGGAGGTTTTTAGAGTAG
PEG3	chr19:57351408-57351561	mat 140	pat 148	CGGGTACGTCGGCGTCGCGAGGTCG	GAGGGTAGTTGGGTATGTTGGTGTTGTG	CCAACAAAATCRACACCAATACTATCCC
GNAS-XL	chr20:57429794-57429979	mat 172	pat 180	GGTAGACGCGCGAGTAGGTCGCG	GTTTTGTTAGGTAGATGTGTGAGTAGGTTGTGG	CAAACRCAAAAACTCCCACTACCCCAACC
GNAS-Ex1A	chr20:57464929-57465107	mat 164	pat 182	GAAATCCCCGCTCTTCCGCGACGCGAACG	CTTCACAAACTTCTCAAAATCCCCACTCTTCCACA	GGGTTATTATGTTGAAGATGGTTATGAAG
WRB	chr21:40757866-40758068	mat 198	pat 205	CCCTACGAACTACACGCACTACGCAAACG	CAAAATCCCTACAAACTACACACACTACACAAACA	GGATAATTTAGAAAAAGTTGAATTTTAAAGGG
NHP2L1	chr22:42078073-42078275	mat 199	pat 209	CATCGTATATAACGTACGAATCGCG	CATATCACCATCATATATAACATACAAATCACA	GTTGTAAAAAAAYGGAAGGAGGAAAAGGTAGGTG

The differentially methylated region (DMR), chromosomal location and primer sequences are provided for each primer set along with the methylated and unmethylated PCR product size.

Supplementary Table 6: MS-SNuPE primers.

Locus	CpG SNuPE Primer Set 1 (hg19)	CpG SNuPE Primer Set 2 (hg19)	PCR Primer-Seq. BT-DNA 5`->3` F [tag]	PCR Primer-Seq. BT-DNA 5`->3` R [tag]	CpG SNuPE Primer Set 1 BT-DNA 5`->3` [t-tag]	CpG SNuPE Primer Set 2 BT-DNA 5`->3` (t-tag)	Elongation Primer Mix 1 [µM]	Elongation Primer Mix 2 [µM]	PCR-Primer Mix [µM] F and R
PLAGL1	chr6:144329491-144329492	chr6:144329594-144329595	[CTTGCTTCCTGGCACGAG]GATAAATGGTAGATGTYGTGGG	[CAGGAAACAGCTATGAC]CAAACCRACTCRAATCTACCTA	[TTTTTTTTTTTTTTTTTTTTTTTTTTTTTTTTTTTTTTTT]CRACRCAACCATCCTCTTAACTACC	[TTTTTTTTTTTTTTTTTTTTTTTTTTTTTTTTTTTTTTTT]ACRAAACCTCCTCCTACCACRTAAC	0.25	0.125	1.5
IGF2R	chr6:160427022-160427023	chr6:160427287-160427288	ATGYGTAGTTGGAGGYGTAT	TACCTCCCCRCACCTTTTAC	[TTTTTTTTTTTTTTTTTTTTTTTTTTTTTTTTTTT]TACCCRCCRCCTCRCCRCRCCCCTC	CRCCTCCCTATACCCTACATACCCC	1.25	0.0875	2.5
GRB10	chr7:50850399-50850400	chr7:50850520-50850521	[CTTGCTTCCTGGCACGAG]YGYGYGTTAGGYGAAYGYGTTAGTAYG	[CAGGAAACAGCTATGAC]TAATCCTAAAATTCCTATTATACTCCAAAAC	[TTTTTTTTTTTTTT]AACRATAACRCRACATCCCACCCTC	[TTTTTTTTTTTTTTTTTTTTTTTTTTTTTTTTTTT]RCTCCTCAAAAACRCCCAATCCCTC	0.125	0.0625	5
MEST	chr7:130132135-130132136	chr7:130132106-130132107	[GATC]TYGTTGTTGGTTAGTTTTGTAYGGTTG	[CAGGAAACAGCTATGAC]CCAACCACACCCCCTCRTTCCCACC	[TTTTTTTTTTTTTTTTTTT]CACCATAACCRCRTTATCCCATACC	[TTTTTTTTTTTTTT]RCCACAACRTTACAAAACACCRAAC	0.125	0.25	2
H19	chr11:2020286-2020287	chr11:2020328-2020329	GGGAGTYGTTTTGTTTGGAG	CAAAACAAACCCCCACATTT	ATATAAATCACCACTACCRCCTCTC	[TTTTTTTTTTTTTTTTTTTTTTTTTTTTT]CAACCACAACCRATTCTATACCATC	0.125	0.125	2.5
KCNQ1OT1	chr11:2721243-2721244	chr11:2721275-2721276	AATTAGTAGGTGGGGGG	CTAAAAAACTCCCTAAAAATC	[TTT]RACRACCRTTCTACCTAAAAACTACRACAAC	[TT]CTCCRACTACCCCCRCCRCTACCRACRTAAC	0.125	0.5	100
MEG3	chr14:101293725-101293901	chr14:101293786-101293787	GYGGGATTTATTGGAGGATT	CCCRAAACCAAAAACCACTA	[TTTTTTTTTTTTTTTTTTTTTTTTT]AAACCACRACRCAAAAACCRACCCC	[TTTTTTTTTTTTTTTTTTT]TCTACRACCACTCCRCAATAAATCC	0.0875	0.25	7.5
SNRPN	chr15:25200519-25200520	chr15:25200653-25200654	GTGGTGTYGYGATAGGTTTT	CTCCTCRCCTCRATCACTAC	[TTTTTTTTTTTTTTTTTTTTTTTTTTTTTTTTTTTTTTTTTTTTT]TCCACCCATATCCCTTACCCACTAC	[TTTTTTTTTTTTTTTTTTTTTTTTTTTTTTTTTTTTTTTTTTTTT]AATACCRATCACTTCACRTACCTTC	0.125	0.25	2.5

The differentially methylated region (DMR), chromosomal location and primer sequences are provided for each primer set.
